# A novel handheld robotic-assisted system for unicompartmental knee arthroplasty: surgical technique and early survivorship

**DOI:** 10.1007/s11701-018-00907-w

**Published:** 2019-02-14

**Authors:** Andrew K. Battenberg, Nathan A. Netravali, Jess H. Lonner

**Affiliations:** 1 Department of Orthopaedic Surgery, Rothman Institute, Thomas Jefferson University, 925 Chestnut Street, Philadelphia, PA 19107 USA; 2 Smith and Nephew, 150 Minuteman Rd, Andover, MA 01810 USA

**Keywords:** Unicompartmental knee arthroplasty, Robotic-assisted surgery, Surgical technique, Survivorship, NAVIO

## Abstract

Technology, including robotics, has been developed for use in unicompartmental knee arthroplasty (UKA) to improve accuracy and precision of bone preparation, implant positioning, and soft tissue balance. The NAVIO™ System (Smith and Nephew, Pittsburgh, PA, United States) is a handheld robotic system that assists surgeons in planning implant positioning based on an individual patient’s anatomy and then preparing the bone surface to accurately achieve the plan. The surgical technique is presented herein. In addition, initial results are presented for 128 patients (mean age 64.7 years; 57.8% male) undergoing UKA with NAVIO. After a mean of follow-up period of 2.3 years, overall survivorship of the knee implant was 99.2% (95% confidence interval 94.6–99.9%). There was one revision encountered during the study, which was due to persistent soft tissue pain, without evidence of loosening, subsidence, malposition or infection. These initial results suggest a greater survivorship than achieved in the same follow-up time intervals in national registries and cohort studies, though further follow-up is needed to confirm whether this difference is maintained at longer durations.

## Introduction

Unicompartmental knee arthroplasty (UKA) is a treatment option for patients with knee osteoarthritis isolated to a single compartment. In comparison with total knee arthroplasty, UKA preserves bone and ligaments and can result in shorter hospitalization and improved patient satisfaction [1]. However, the success of UKA depends on a variety of factors including patient selection, implant design, component alignment and fixation, and soft tissue balance [2–6]. It is challenging to obtain correct implant alignment using standard instrumentation, which has been shown to result in outliers beyond 2° of the planned alignment in as many of 40–60% of cases [7, 8].

Robotic-assisted systems are designed to improve a surgeon’s ability to accurately achieve the desired limb and component alignment, optimize soft tissue balance, control the joint line and restore normal knee kinematics in UKA [9–16]. Several types of robotic-assisted surgery have been available in orthopaedics for nearly 25 years. Current robotic-assisted systems often use various navigation principles augmented with the technology of robotic bone preparation, allowing the surgeon to conduct a UKA based on preoperative 3D images or image-free intra-operative planning [17]. It is estimated that 15–20% of UKA surgeries in the United States are performed with robotic assistance [18].

In orthopaedics, robotic assistance can be used to perform specific tasks in achieving a plan according to preoperative data. The level of involvement of the robotics can vary and three main categories have been described: passive, semi-active, and active robotics [19]. Passive systems, also known as computer-assisted navigation systems, provide the surgeon with perioperative recommendations for guiding positioning, but this and bone resection are all done under direct control of the surgeon without true robotic assistance. Semi-autonomous robots are tactile feedback systems that augment the surgeon’s ability to control and manipulate the robotic tool, typically by restricting the resection volume by haptic constraint or by controlling the cutting tool motion or exposure. Finally, active robotic systems perform a surgical task without direct surgical tool manipulation by the surgeon, other than inputting the initial surgical plan.

The NAVIO™ System (Smith and Nephew, Pittsburgh, PA, USA) is a next-generation semi-autonomous tool that uses handheld miniaturized robotic-assisted instrumentation that the surgeon manipulates in 6° of freedom, but restricts cutting to within the confines of the pre-designated resection area of the patient’s bone. This article describes the surgical technique for using the NAVIO system for UKA, as well as early implant survivorship results associated with its use.

## Methods

This was a retrospective, multi-center, cohort study to collect 2-year survivorship data on patients who had undergone NAVIO System-assisted UKA. The surgical technique is described in detail below. The NAVIO system was used for all patients to plan the positioning of the implants and to prepare the bone surfaces prior to implantation. These patients represent the initial series of NAVIO-assisted UKA procedures performed by five surgeons to eliminate the potential for any selection bias. If the patients had not completed a 2-year follow-up visit, their survivorship and adverse event status was collected prospectively. For patients that had already completed a 2-year visit at the time of enrollment, their last follow-up was included retrospectively. Informed consent was obtained from all individual participants included in the study.

For the analysis of survivorship, the cumulative proportion (or percentage) of subjects with implant survivorship at 2+ years (96 weeks), the following hypothesis was performed using the safety population:

H0 (null): *S*(*t*) = *π* ≤ (*π*_0_ − *δ*) versus

Ha (alternate): *S*(*t*) = *π* > (*π*_0_ − *δ*),

where *π* = expected percentage of subjects with implant survivorship at 2 years, *π*_0_ = historical control implant survivorship (95.7%, from the Australian registry [23]) and *δ* = margin of non-inferiority (7%).

Subjects who completed the study without a revision were censored at their last known date in the study while those who prematurely discontinued from study as a result of death or for any other reason were censored at the date this event occurred. Any subject lost to follow-up was censored at their last known contact date. The Kaplan–Meier estimate for implant survivorship is presented with the corresponding two-sided 95% Confidence Intervals (CIs).

### Surgical technique

**Fig. 1:**
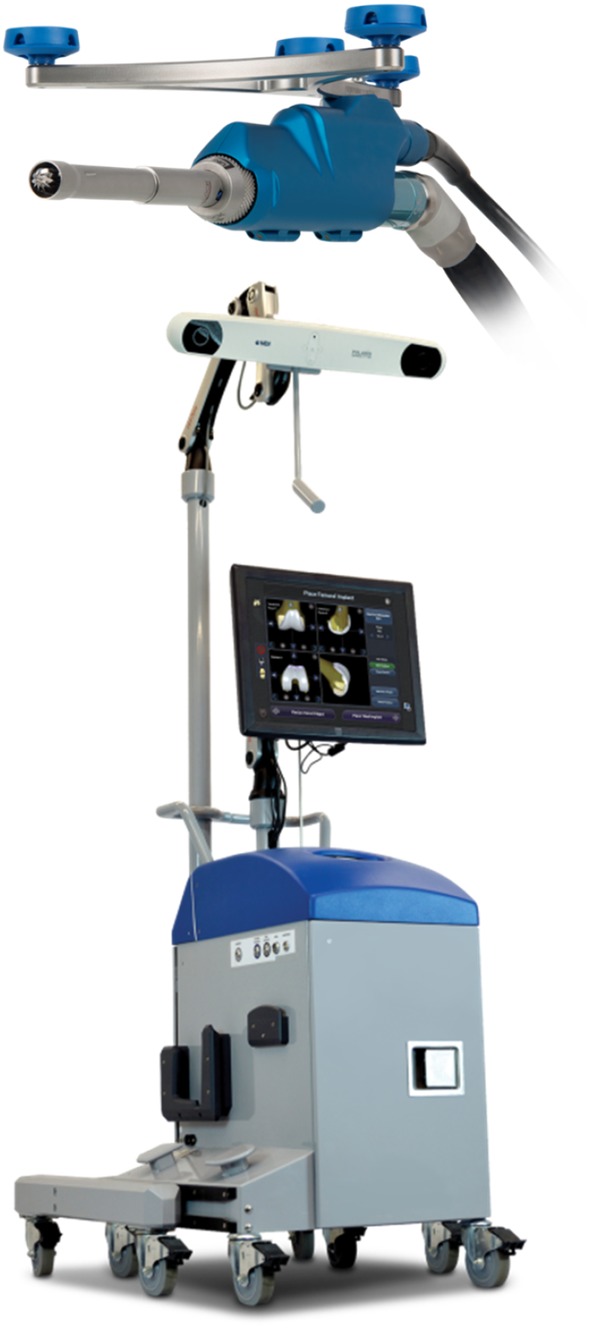
The NAVIO surgical system and handpiece

The NAVIO system consists of several components (Fig. 1). A tracking system and stand uses infrared cameras to determine the position of reflective trackers in space. The accompanying cart contains the electronic control system, an electrical system integration unit, a computer, an uninterruptible power supply, and a touchscreen monitor, which serves as the primary user interface. There are two foot-control pedals, one of which serves as an alternative user interface to the touchscreen monitor, and the other to control the Anspach drill that runs the motorized robotic bur that prepares the bone. Lastly, a handpiece controls the position of the Anspach drill and bur relative to the position of the guard and the desired profile of the bone being cut.

The NAVIO surgical system is indicated for use in knee procedures in which the use of stereotactic surgery may be appropriate, and where reference to rigid anatomical bony structures can be determined. This includes UKA, patellofemoral arthroplasty (PFA) and total knee arthroplasty (TKA). The overall surgical procedure is divided into six distinct steps described below in the following sections.

### Step one: patient and system setup

In the operating room, the cart containing the NAVIO computer and monitor is positioned next to the operating table. After incision, all peripheral osteophytes are removed so that joint stability can be adequately assessed. Tracking arrays are attached to both the femur and the tibia using a two-pin bi-cortical fixation system. On the tibia, the pins are placed percutaneously inferior to the tibial tubercle on the medial side of the tibial crest. On the femur, the screws are placed superior to the patella. Additionally, small checkpoint pins are placed in the femur and tibia, which are used to determine if the trackers have moved relative to the bone at various points during the procedure.

### Step two: registration

**Fig. 2:**
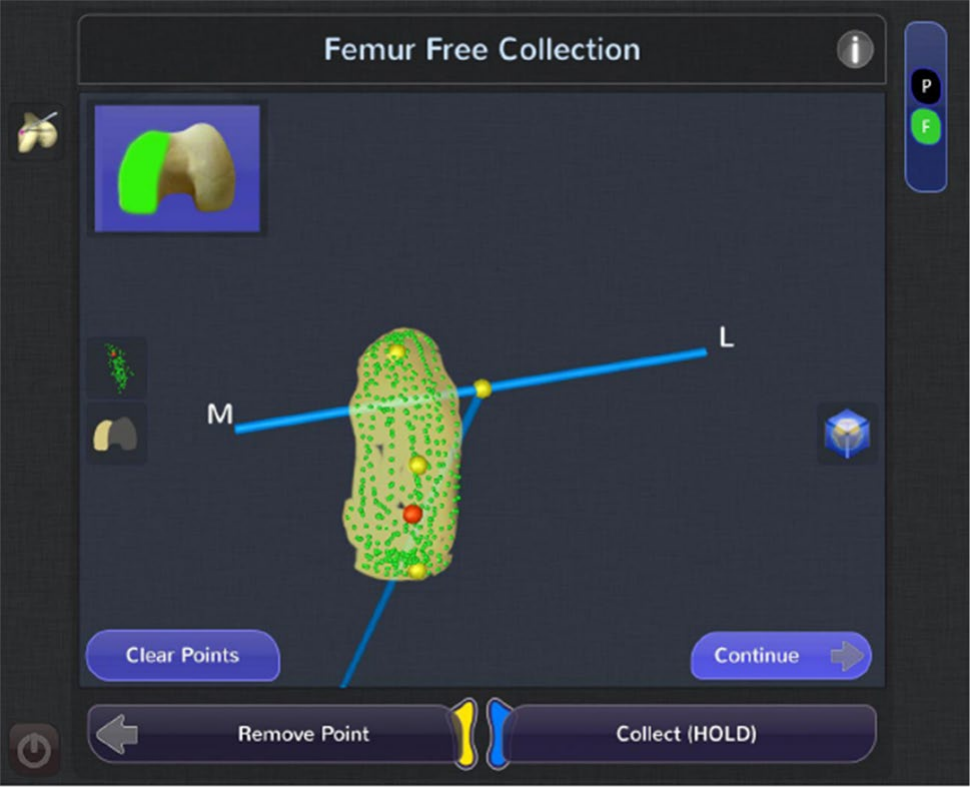
Surface model creation of the femur during registration

The NAVIO system is different from other robotic systems in that it does not require any preoperative imaging for planning; sparing the patient exposure to radiation associated with computed tomography scans [21]. The entire process of defining the patient’s anatomy and planning workflow is performed intra-operatively. This technique of registering the patient’s anatomy is done using a localization of anatomic landmarks and a surface “painting” technique to create a 3D virtual model of the patient’s anatomy (Fig. 2). These landmarks are collected using a point probe, which contains a tip that is tracked using the infrared cameras. This probe is used to collect points and “paint” the surface of the bone and/or articular surfaces by holding down the foot pedal.

The surgeon is guided to collect points on the medial and lateral malleoli to register the ankle center. The surgeon then takes the leg through a rotation about the hip to calculate the hip center. The leg is then placed in full extension and put under slight compression to capture any varus/valgus deformity and flexion contracture. The mechanical axis of the limb is then calculated by the computer system, based on these registered points. The user then takes the knee through a range of motion to maximum flexion while keeping the knee joint in contact, establishing the rotational axis of the limb, from which femoral component rotation is derived. Afterwards, varus or valgus stress is applied to tension the soft tissues on the sides of the knee through a full range of flexion to plan the desired soft tissue laxity. This helps the surgeon plan for implant positioning and volume bone resections, taking into account “virtual” soft tissue laxity prior to making any cuts. Next, the user collects a number of reference points on the femur and tibia, demarcating the boundaries of the hemi-plateau or hemi-condyle, and defining the mechanical axes of the femur and tibia. Surface mapping is then completed by “painting” the entire condylar surface while holding down the foot pedal and creating a three-dimensional virtual model of the involved knee compartment. Once both the femur and tibia are registered, prosthesis planning can begin.

### Step three: prosthesis planning

**Fig. 3:**
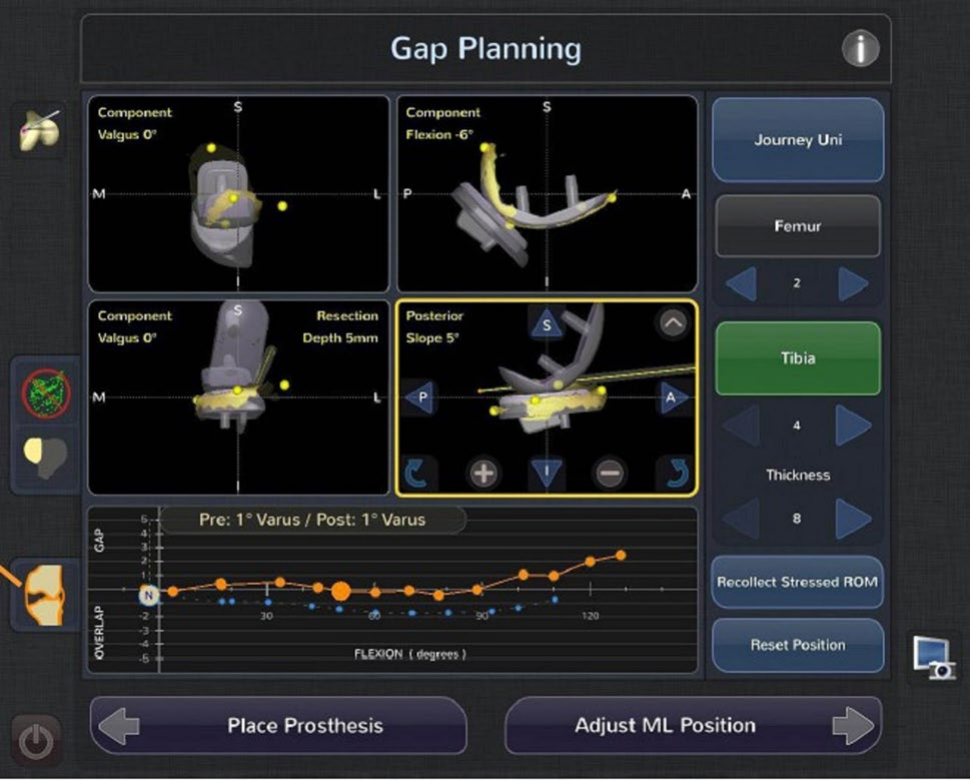
Planning screen to show predicted gaps throughout a range of flexion

In the planning stage, the system provides the user with a virtual reconstruction of the patient’s femoral and tibial anatomy, soft tissue ligament tension, and joint balance. The first step of planning is the initial sizing and placement of the implants, which is performed automatically by the NAVIO software through the use of the landmarks and the painted bone surfaces, and then adjusted by the surgeon. The surgeon can then assess the depth of resection and alignment with respect to the mechanical axis. The software provides the user with the expected laxity balance throughout a range of flexion and extension (Fig. 3). The goal is to adjust the implant positions and orientations such that the gaps in extension and flexion are balanced, with roughly 1–2 mm of laxity between the components through a full arc of motion, and avoiding over-correction of alignment into the opposite compartment. To achieve adequate balance, adjustments in implant flexion, rotation, translation, varus/valgus, and depth can be made. Once the surgeon is satisfied with the implant positions and soft tissue balance, the next step is preparation of the bone surfaces using the NAVIO robotic handpiece.

### Step four: robotic-assisted bone cutting

**Fig. 4:**
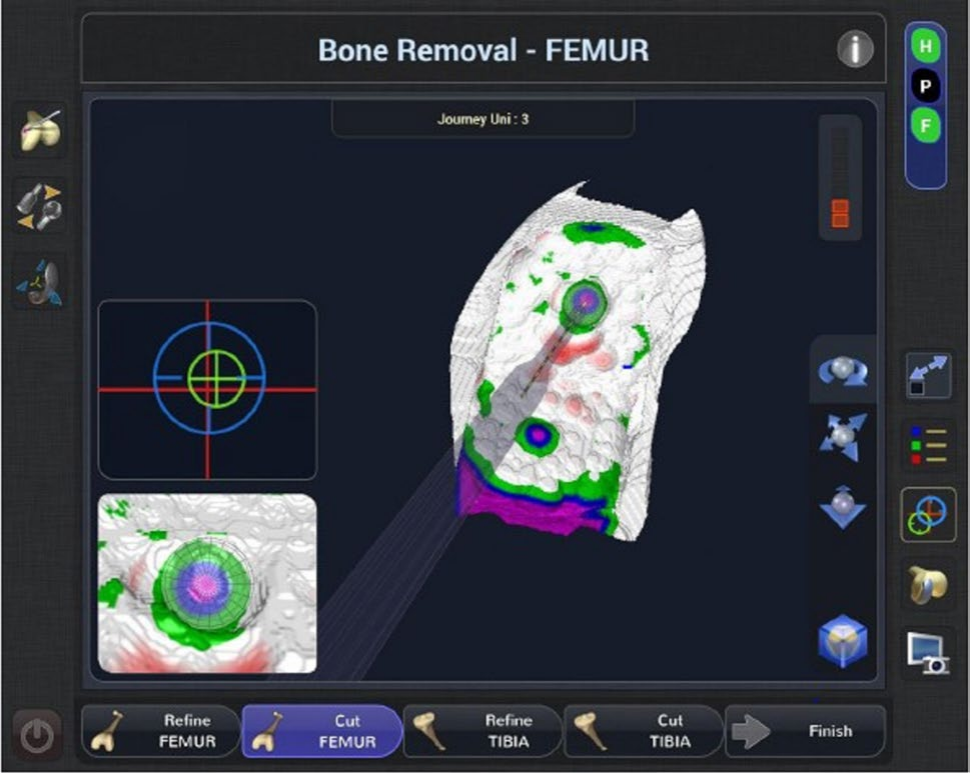
Screen guidance during bone preparation showing the remaining bone to be removed

The NAVIO handpiece is a semi-autonomous robotic tool in which the surgeon can move the handpiece freely in space. However, the cutting action is disabled when the tip is outside the designated cutting space. This can be done via two different methods of robotic control. The first method is termed “exposure control,” in which the system retracts the bur within a protective guard when the motorized bur is outside the designated cutting space. The second method is termed “speed control”, in which the speed of the bur is automatically slowed and ultimately stopped when the cutter reaches the edge of the cut planes. For the UKA application, the NAVIO system is used to prepare all of the bony surfaces including the tibial and femoral planar surfaces and peg holes (Fig. 4).

### Step five: trial reduction

**Fig. 5:**
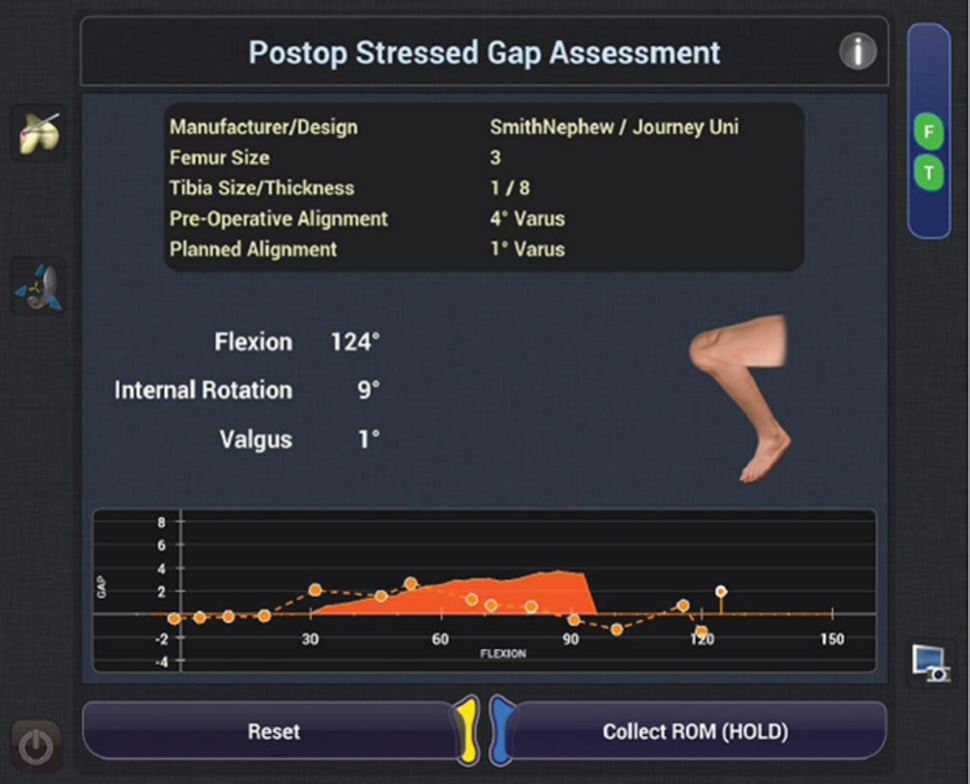
Post-operative gap assessment under stress throughout a range of flexion

After the bone surfaces are prepared to the satisfaction of the surgeon, the wound is irrigated and dried. The surgeon then manually provisionally inserts the trial components ensuring appropriate alignment and balance through a full range of motion. The system displays the achieved coronal alignment and laxity of the knee and allows a comparison with the initial plan created in the planning stage. The system then prompts the user to assess the post-operative gap balance throughout flexion in both the medial and lateral compartments (Fig. 5).

Adjustments can be made if balance or alignment modifications are deemed appropriate. This is done with ease by returning to the planning and bone removal stage and adjusting appropriate parameters, such as slope or depth of resection of either, or both, components. The surgeon can then make any re-cuts using either exposure or speed control mode if there are any changes to the plan needed to achieve a different soft tissue balance. When the surgeon is satisfied with the final results, manual implantation proceeds using the surgeon’s standard methods.

## Initial results

Patients were followed for a mean of 2.3 years to determine if the UKA had been revised for any reason. The 128 participants had a mean age at surgery of 64.7 years (median 64; range 45–92; standard deviation 9.6), with the majority (89 subjects; 69.5%) 60 years of age or older and male (74 subjects; 57.8%). They had a mean body mass index of 30.3 kg/m^2^ (median 29.7; range 19.9–45.9; standard deviation 5.4), with a slight majority (66 subjects; 52%) in the body mass index category of < 30 kg/m^2^. UKA was performed on the medial compartment in 124 subjects (96.9%) and on the lateral compartment in four subjects (3.1%).

Overall survivorship of the knee implant in these robotic-assisted cases at 2 years (96 weeks) was 99.2% (95% confidence interval 94.6–99.9%), which was non-inferior when compared to the reference survival rate of 95.7% from the Australian registry [20]. There was one revision encountered during the study, which was due to persistent soft tissue pain, without evidence of loosening, subsidence, malposition or infection. This revision occurred at 218 days post-op in a male less than 60 years old with a body mass index < 30 kg/m^2^.

There were 4 (3.1%) patients who reported adverse events that were possibly or definitely related to the NAVIO System, and 16 (12.5%) who reported adverse events that were possibly or definitely related to the knee implant. The adverse events that may have been related to NAVIO included one case each of stress shielding, incision pain with deep flexion and anterior knee pain and swelling, synovial hypertrophy with quadriceps atrophy and knee pain, and persistent soft tissue pain. The adverse events that may have been related to the implant included one case each of synovial hypertrophy with quadriceps atrophy and knee pain, persistent soft tissue pain, general soreness, femoral osteolysis, and anterior knee pain and incisional pain with deep flexion, and 11 cases of non-progressive radiolucent lines.

## Discussion

The NAVIO system is a unique robotic-assisted technology for use in partial and total knee arthroplasty. The system design optimizes planning for joint replacement, with the benefits of no additional radiation from CT, and a handheld robotic tool resulting in precision cuts. There is a clear importance to achieving soft tissue balancing in UKA procedures and the NAVIO system incorporates ligament laxity throughout a full range of motion during the planning stage. The combination of precise component alignment, kinematic restoration, and ligament balance, likely account for the high durability observed in our pilot study. By utilizing the software that predicts the ligament laxity based on the surgical plan, the surgeons have the ability to optimize implant placement to fully take into account these soft tissue considerations.

Although robotics has been used to assist UKA for several years, there is limited data available on how its use may affect survivorship. Early studies have shown precision and elimination of alignment errors that are comparable to those observed with robotic systems that require preoperative CT scans [14, 16, 22]. Further, the NAVIO system has been shown to achieve limb alignment within 1–2° of the plan with more accurate restoration of the joint line compared to conventional methods [13, 23]. Ours is the first to look at the 2-year revision rates for NAVIO robotic-assisted UKA in a retrospective study of 128 patients at 5 sites within the United States.

At 2 years there was only one revision resulting in a survivorship rate of 99.2%. This was despite these cases being the surgeon adopters first cases, during a period of the so-called learning curve. The survivorship rate of conventional UKA at the same follow-up period is well reported in the literature and in several national registries. By way of comparison, 2-year survivorship for conventional UKA was 95.7% in the Australian registry [20], 96.3% in the New Zealand registry [24], and 96% in the Swedish registry [25]. Further, several cohort studies also report 2–3-year survivorship and these are presented in Table 1. It can be seen that survivorship in these studies ranges from 94.7 to 98.0%, which are lower than the reported durability found in our study.

**Table 1:** Survivorship reported in cohort studies reporting 2- to 3-year follow-up

Cohort study	# Cases	Survivorship at 2–3-year follow-up (%)
Eickmann et al. [26]	411	96.0
Hamilton et al. [27]	517	97.0
Liebs and Herzberg [28]	401	94.7
Lim et al. [29]	400	97.4
Pandit et al. [30]	1000	98.0
Vorlat et al. [31]	149	97.8
Yoshida et al. [32]	1279	98.3

In this multicenter study, UKA with NAVIO robotics assistance was shown to have a high survivorship at a short-term follow-up. These initial results suggest a greater survivorship than achieved in the same follow-up time intervals in national registries and cohort studies, though further follow-up will be needed to confirm whether this difference is maintained at longer durations. Nonetheless, these early results indicate that the NAVIO system has strong potential to improve patient outcomes.
